# Occlusion and Mastication

**Published:** 1895-12

**Authors:** W. H. H. Barker

**Affiliations:** Huron, South Dakota.


ARTICLE V.

### OCCLUSION AND MASTICATION.

DR W. H. H. BARKER, HURON, SOUTH DAKOTA.

In occlusion every tooth except the lower central incisor and the third molar impinges or rests its occluding surface on at least two of its fellows in the apposing arch, and each in its own arch, except the third molar, impinges on its mesial and distal surfaces with the same number of of its fellows in its own arch.

On these two facts are based many laws which should never be ignored in any operation in the oral cavity, so far at least as the teeth are concerned.

One of these laws, and the one that perhaps is the least considered, is that of facial expression. There cannot be a perfect face where there is a malocclusion. No part of the face is so subject to change as that bounded by the maxillaries. Infancy, youth and old age proclaim these truths.

Often malocclusion prevents clear, distinct and perfect articulation, and allows a disarrangement of the whole apparatus. Elongation, or protrusion are marked features, and most seriously interferes with the free movements of the lips and tongue, and these in turn interfere with enunciation to such an extent that in many cases the voice is marred beyond recognition and almost beyond redemption This is a feature that calls loudly for remedy at the hands of the dental surgeon, and should be much less ignored than it now often is.

It is a physiological law that the full development of an organ and its maintenance in a normal healthy condition is dependant on its use. Nature allows no drones, physiologically, and hence the work is designed for an organ must be performed by it, or it suffers in consequence.

The dental organs are no exception to the rule. They are made for work, and hard work of the most severe and constant kind. That this work may be done rightly, and to its full extent, there must be no break in either arch, and occlusion must be perfect and complete. Malocclusion does not allow of either normality or healthfulness in the teeth, either as a unit, or as an aggregation; work is their like, and antagonism their salvation. In malocclusion, both, to some extent, are wanting, their office abrogated, and they must pay the penalty. Disease readily attacks a single member, or the whole of that arch, if not put to service, and the role of nature, is to expunge what is of no service. Among the many causes of malocclusion may be noted irregularity, a most fruitful source, happily in a large number of cases remedial. It is seen in its worst form in a crowded condition, and is the result of a want of correspondence in the size of the teeth and jaws. Then comes the lack of the proper number, and results usually from loss of caries or extraction after the organ has developed and taken its place in the arch. Next, the wasting of the gums and alveolar processes from mercurial salivation, pyorrhœa alveolaris, the impingement of salivary and sanguinary calculus; and other diseases, causing the teeth to change their positions; and to assume new ones.—*Dental Review.*

